# MCL1 inhibition: a promising approach to augment the efficacy of sorafenib in NSCLC through ferroptosis induction

**DOI:** 10.1038/s41420-024-01908-5

**Published:** 2024-03-14

**Authors:** Chao-Yuan Huang, Li-Ju Chen, Chi-Shuo Chen, Cheng-Yi Wang, Shiao-Ya Hong

**Affiliations:** 1 Division of Radiation Oncology, Department of Oncology, National Taiwan University Hospital, College of Medicine, National Taiwan University, Taipei, 100229 Taiwan; 2 Department of Biomedical Engineering and Environmental Sciences, National Tsing Hua University, Hsinchu, 300044 Taiwan; 3 Department of Internal Medicine, Cardinal Tien Hospital and School of Medicine, College of Medicine, Fu Jen Catholic University, New Taipei, 231009 Taiwan; 4 Department of Biotechnology and Laboratory Science in Medicine, National Yang Ming Chiao Tung University, Taipei, 112304 Taiwan

**Keywords:** Non-small-cell lung cancer, Cell death

## Abstract

Ferroptosis, an iron-dependent form of regulated cell death, plays a crucial role in modulating the therapeutic response in non-small cell lung cancer (NSCLC) patients. Studies have identified the signal transducer and activator of transcription 3 (STAT3) and myeloid cell leukemia-1 (MCL1) as potential targets for sorafenib, which exhibits activities in inducing ferroptosis. However, the role of STAT3-MCL1 axis in sorafenib-induced ferroptosis in NSCLC is still unclear. This study provided evidence that ferroptosis is a critical driver of sorafenib-induced cell death in NSCLC, supported by the accumulation of lipid peroxidation products, indicative of oxidative stress-induced cell death. Additionally, both in vitro and in vivo experiments showed that ferroptosis contributed to a significant portion of the anti-cancer effects elicited by sorafenib in NSCLC. The noticeable accumulation of lipid peroxidation products in sorafenib-treated mice underscored the significance of ferroptosis as a contributing factor to the therapeutic response of sorafenib in NSCLC. Furthermore, we identified the involvement of the STAT3/MCL1 axis in sorafenib-induced antitumor activity in NSCLC. Mechanistically, sorafenib inhibited endogenous STAT3 activation and downregulated MCL1 protein expression, consequently unleashing the ferroptosis driver BECN1 from the BECN1-MCL1 complex. Conversely, there is an augmented association of BECN1 with the catalytic subunit of system Xc^−^, SLC7A11, whose activity to import cystine and alleviate lipid peroxidation is hindered upon its binding with BECN1. Notably, we found that MCL1 upregulation correlated with ferroptosis resistance in NSCLC upon sorafenib treatment. Our findings highlight the importance of sorafenib-triggered ferroptosis in NSCLC and offer a novel strategy to treat advanced NSCLC patients: by downregulating MCL1 and, in turn, predispose NSCLC cells to ferroptosis.

## Introduction

About 85% of all lung cancer cases are classified as non-small cell lung cancer (NSCLC), which is the leading cause of cancer-related deaths globally. Even with recent improvements in outcomes and a decrease in death rates, the five-year survival statistic remains at a mere 19.3% [1]. For decades, chemotherapy based on cisplatin has been the leading standard of care in the clinical management of advanced NSCLC without particular actionable molecular targets [2, 3]. Over the past few years, an increasing amount of new molecular alterations, including some oncogenes and tumor suppressor genes, has been identified in NSCLC. Many of those molecular alterations represent novel predictive biomarkers or actionable targets for cancer therapy. Hence, targeted therapies have gradually become the first-line choice for NSCLC harboring specific genetic disorders, such as *EGFR* mutations and *ALK* translocations [4]. Unfortunately, drug resistance to chemotherapy or targeted therapies inevitably develops after treatment. Immunotherapies by exploiting immune checkpoint inhibitors have emerged and changed the treatment landscape for various cancers. Although the advent of immune checkpoint inhibitors (ICIs) has been encouraging, the overall response rates are unsatisfactory, with a huge portion of NSCLC patients not receiving therapeutic benefits from ICIs. Therefore, new therapeutic strategies are urgently needed for the treatment of NSCLC.

Ferroptosis, an iron-dependent form of regulated cell death, is distinct from other known forms of cell death, such as apoptosis, necroptosis, pyroptosis, and autophagy [5]. The primary characteristics of ferroptosis include the shrinking of mitochondria with thicker membrane structures, along with either a decrease or loss of mitochondrial cristae, and the breaking of the outer mitochondrial membranes [6]. Biochemically, the perturbation of the intracellular redox balance and subsequent iron-dependent peroxidation of polyunsaturated fatty acids (PUFAs) in cell membrane phospholipids result in the lethal accumulation of lipid-based reactive oxygen species (ROS) due to the inactivation of glutathione peroxidase 4 (GPX4) [7]. Ferroptosis is linked to a variety of pathological conditions, including neurodegenerative disorders, carcinogenesis, stroke, intracerebral hemorrhage, traumatic brain injury, and ischemia-reperfusion injury [8–11]. Ferroptosis has also been demonstrated as a critical cell death mechanism in various cancers, such as hepatocellular carcinoma (HCC), renal cell carcinoma (RCC), ovarian cancer, pancreatic carcinoma, diffuse large B cell lymphoma (DLBCL), and non-small cell lung cancer (NSCLC) [12]. Recently, evidence has shown that ferroptosis plays a vital role in mediating the sensitivity to, and attenuating drug resistance of, chemotherapy and EGFR-targeted therapies [13–20]. Therefore, promoting ferroptosis may be a promising approach to benefit NSCLC patients from current treatments.

Experimental compounds, notably erastin, RSL3, and buthionine sulfoximine, or clinically approved medications (e.g., sulfasalazine, sorafenib, and artesunate) have been mentioned to induce ferroptosis in cancer [21]. Sorafenib, previously known to inhibit the kinase activities of both C-RAF and B-RAF, and to target the vascular endothelial growth factor receptors (such as VEGFR-2 and VEGFR-3) and platelet-derived growth factor receptors (namely PDGFR-β and KIT) [22], is approved for the clinical treatment of advanced renal cell carcinoma, thyroid cancer, and hepatocellular carcinoma [23, 24]. Accumulating studies have also demonstrated that sorafenib exhibits certain levels of therapeutic effects in other cancer types, including NSCLC [25]; however, only a subset of NSCLC patients benefit from clinical use of sorafenib [26]. Interestingly, Li et al. suggested that sorafenib triggers ferroptosis in cisplatin-resistant NSCLC cells by inhibiting the NRF2/xCT pathway [27]. Therefore, deciphering the molecular mechanisms of sorafenib-induced ferroptosis in NSCLC could pave the way for harnessing and optimizing ferroptosis-centric therapies for NSCLC and offer a scientific basis for incorporating ferroptosis induction in the combination treatment regimens.

Signal transducer and activator of transcription 3 (STAT3) has been identified as a potential target for sorafenib [28]. STAT3 is a transcription factor that mediates nuclear gene expression to regulate a variety of cellular functions, and its constitutive activation contributes to the malignant phenotypes of cells [29]. STAT3 inactivation triggered by sorafenib has been shown to elicit different types of cancer cell death including apoptosis [28, 30, 31] and autophagy [32], possible via myeloid cell leukemia-1 (MCL1) downregulation. However, the role of the STAT3/MCL1 signaling axis in sorafenib-induced ferroptotic death in NSCLC remains unclear. We sought to determine whether and how STAT3/MCL1 signaling axis is involved in sorafenib-induced ferroptosis and to identify potential biomarkers for selecting NSCLC patients who may benefit from treatment with ferroptosis-inducing agents.

## Results

### Sorafenib induces ferroptosis in specific non-small cell lung cancer cell lines

**Fig. 1:**
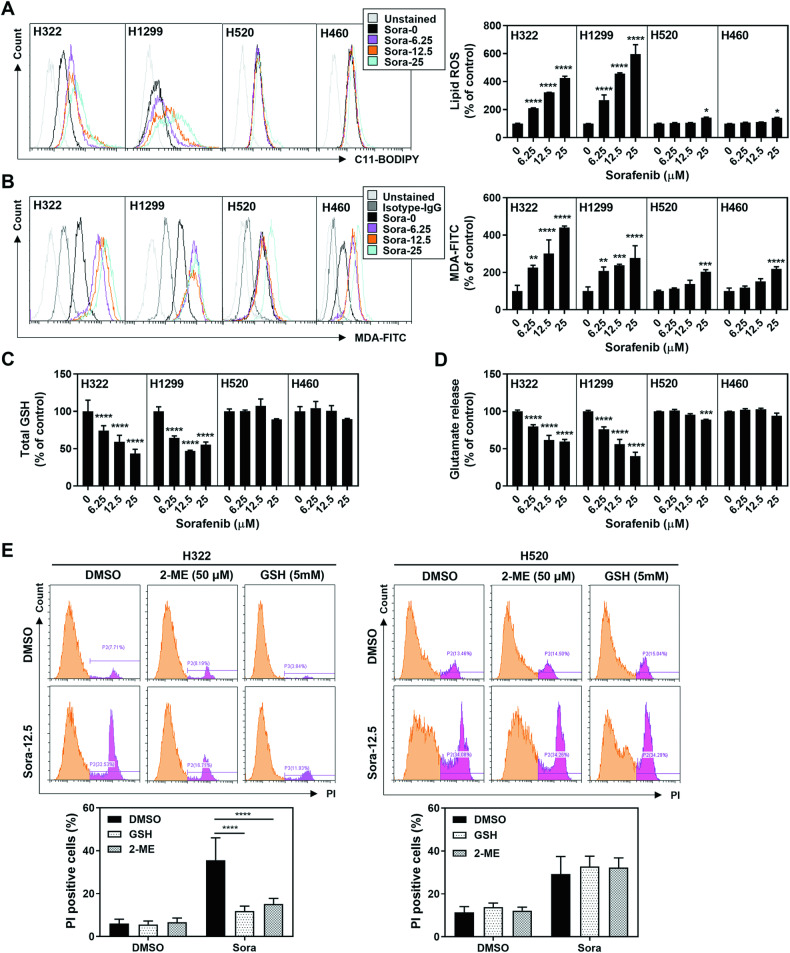
Sorafenib induces ferroptosis in certain types of non-small cell lung cancer (NSCLC) cells. Ferroptosis-sensitive (H322 and H1299) and ferroptosis-resistant (H520 and H460) cell lines were treated with indicated doses of sorafenib for 16 h. The levels of lipid peroxidation were determined by performing C11-BODIPY (**A**) and MDA (**B**) assay. Intracellular GSH content was determined utilizing the Glutathione assay (**C**). Inhibition of system Xc^−^ activity was quantified using the glutamate release assay (**D**). Viability of cells in response to sorafenib was assessed by the PI-staining assay (**E**). All results represent the mean ± SD from three independent biological replicate experiments. *p < 0.05; **p < 0.01; ***p < 0.001; ****p < 0.0001.

To examine the ferroptosis-inducing capacity of sorafenib in NSCLC, we employed two ferroptosis inhibitors, Liproxstatin-1 (Liprox) and deferoxamine (DFO), in a panel of 12 distinct NSCLC cell lines. The results revealed that the cell death triggered by sorafenib was effectively counteracted by these ferroptosis inhibitors in certain NSCLC cell types, as detailed in Table 1. To further verify whether sorafenib-induced cell death could be attributed to ferroptosis, we measured the accumulation of lipid-based reactive oxygen species (ROS) and malondialdehyde (MDA), both recognized hallmarks of ferroptosis. Remarkably, H322 and H1299 cells treated with sorafenib exhibited dose-dependent increases in both metrics (Fig. 1A, B), indicating a specific role for sorafenib as a ferroptosis inducer in these NSCLC cells. Moreover, we observed that sorafenib treatment led to a significant reduction in total glutathione (GSH), a crucial molecule that safeguards tissues against oxidative damage (Fig. 1C). Results also showed that sorafenib treatment hindered the cysteine-glutamate exchange, which is part of the anti-peroxidation defense mainly facilitated by the antiporter system Xc^−^, as evidenced by the declined glutamate release (Fig. 1D). In contrast, resistant cell lines displayed only slight changes in these metrics at the high dose of sorafenib (Fig. 1A–D). Crucially, the induction of ferroptotic cell death by sorafenib in sensitive cell lines could be effectively reversed by introducing reduced forms of GSH or 2-ME, but this was not the case in resistant lines (Fig. 1E). These findings provide strong evidence that sorafenib induces ferroptosis in certain types of NSCLC and underscore the contribution of ferroptosis to sorafenib-induced cell death in NSCLC.

**Table 1:** Sorafenib-induced ferroptotic death in NSCLC cell lines.

Cell line	Sorafenib + DMSO (mean ± SD)	Sorafenib + Liprox-1 (mean ± SD)	Sorafenib + DFO (mean ± SD)	*p*-val^a^	*p*-val^b^
*Ferroptosis sensitive*					
H322	81.10 ± 7.53	19.45 ± 6.10	29.36 ± 8.53	<0.0001	<0.0001
H1299	86.72 ± 7.13	21.55 ± 6.03	34.97 ± 8.96	<0.0001	<0.0001
H358	89.56 ± 8.64	22.90 ± 9.54	34.88 ± 6.59	<0.0001	<0.0001
SW1573	77.18 ± 11.30	16.27 ± 4.72	27.38 ± 5.95	<0.0001	<0.0001
H1703	81.80 ± 0.48	17.73 ± 2.54	30.19 ± 1.01	<0.0001	<0.0001
PC9	95.98 ± 0.55	29.27 ± 2.51	29.13 ± 1.92	<0.0001	<0.0001
*Ferroptosis resistant*					
H292	66.79 ± 7.73	71.03 ± 1.38	53.60 ± 13.07	0.9552	0.1723
H520	46.99 ± 9.84	58.15 ± 3.80	65.68 ± 6.04	0.1427	0.0068
H460	97.59 ± 0.09	97.90 ± 0.24	97.77 ± 0.39	>0.9999	>0.9999
H1437	79.17 ± 5.07	86.78 ± 0.93	81.49 ± 4.90	0.0876	0.9332
H1975	16.80 ± 2.82	24.77 ± 0.82	23.43 ± 2.74	0.0053	0.0199
HCC827	42.16 ± 1.24	42.79 ± 9.96	49.31 ± 2.49	>0.9999	0.4237

Note: Sorafenib-induced cell death was assessed by employing PI-staining. Values indicate the percentage of cell death and are given as the mean ± SD from three independent experiments conducted in triplicates.

^a^DMSO v.s. Liprox-1.

^b^DMSO v.s. DFO.

### Sorafenib exerts antitumor effects, at least partially, by promoting ferroptosis in vivo

**Fig. 2:**
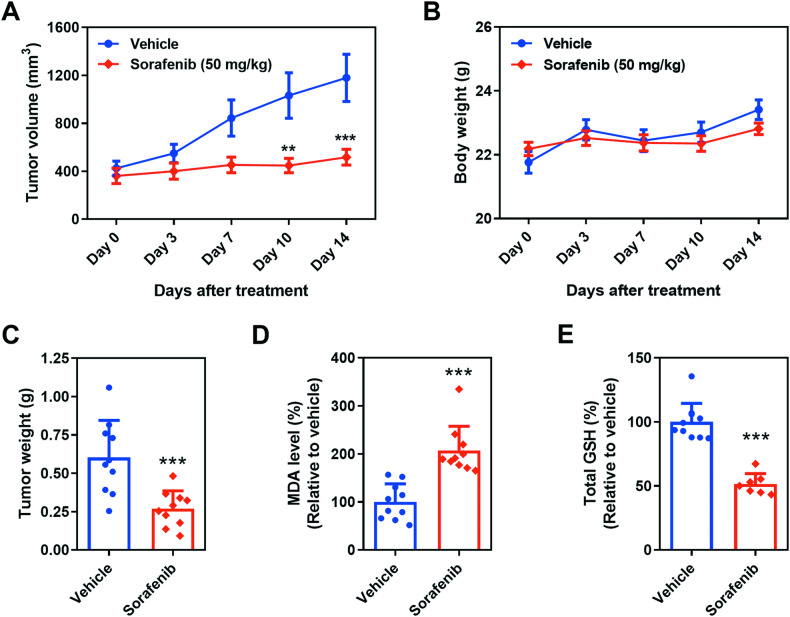
Sorafenib exhibits anti-tumor effects in ferroptosis-sensitive NSCLC cells in vivo. The tumor growth curve (**A**) and body weight changes (**B**) in H322 xenograft mice were monitored over a specific time course. Data are presented as the mean ± SEM (*n* = 10 for each group). The tumor weight of xenografts was assessed at the completion of the designated treatments (**C**). Lipid peroxidation (**D**) and total GSH levels (**E**) in tissue extracts in response to sorafenib treatment were expressed as a percentage relative to the control group. Results for **C**–**E** are represented as the mean ± SD (*n* = 7–10). **p < 0.01; ***p < 0.001.

To investigate the potential of sorafenib in inducing ferroptosis in NSCLC within an in vivo setting, we established the H322 xenograft model in mice. Our results showed a significant reduction in both tumor volume (Fig. 2A) and tumor weight (Fig. 2C) in mice receiving sorafenib, compared to the control group. Importantly, these therapeutic benefits were achieved without significant adverse impact on the body weight of the treated mice (Fig. 2B), suggesting a favorable safety profile for clinical translation. Moreover, sorafenib treatment led to a notable increase in the levels of malondialdehyde (MDA), an indicator of lipid peroxidation, in the tumor microenvironment (Fig. 2D). Simultaneously, the total levels of reduced glutathione (GSH), a major cellular antioxidant, were found to be reduced (Fig. 2E). These findings collectively provide solid evidence for the involvement of ferroptosis, which is characterized by a surplus of lipid peroxidation, in NSCLC tumor regression following sorafenib treatment.

### Ferroptosis is the major types of sorafenib-induced cell death

**Fig. 3:**
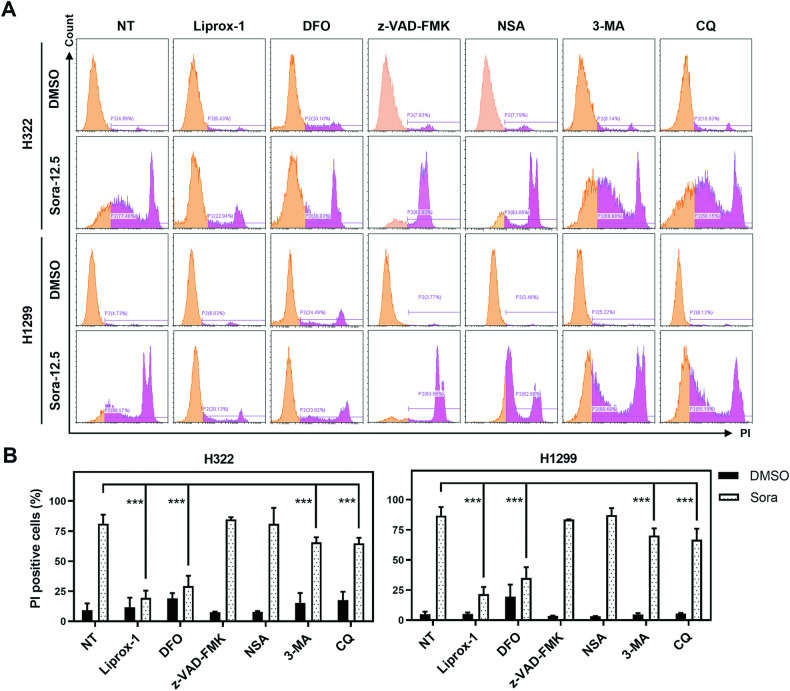
Ferroptosis is the principal mechanism driving sorafenib-induced cell death in ferroptosis-sensitive NSCLC cells. Cell death of H322 and H1299 cells in response to sorafenib treatment, using the indicated inhibitors, was assessed by flow cytometry following PI staining (**A**). The bar graphs depict the mean ± SD obtained from three independent biological replicate experiments (**B**). ***p < 0.001.

To assess the contribution of ferroptosis in sorafenib-induced cell death in H322 and H1299 cells, we compared the effects of ferroptosis inhibitors, Liprox-1, and DFO, with apoptosis and necroptosis inhibitors, Z-VAD-FMK and necrosulfonamide (NSA), respectively. Surprisingly, we noted that sorafenib-induced cell death was not effectively mitigated by Z-VAD-FMK and NSA (Fig. 3A, B), suggesting that apoptosis and necroptosis might not be the primary drivers of cell death in sorafenib-treated NSCLC cells. We also evaluated the impact of autophagy on sorafenib-induced NSCLC cell death by employing autophagy inhibitors, 3-methyladenine (3-MA) and chloroquine (CQ). Interestingly, our data revealed that while autophagy inhibitors 3-MA and CQ provided certain levels of protection against sorafenib-induced cell death, they did not alleviate the cytotoxic effects of sorafenib (Fig. 3A, B) as pronouncedly as ferroptosis inhibitors did. These findings highlight the predominant role of ferroptosis in sorafenib induced NSCLC cell death.

### STAT3/MCL1 axis involves in sorafenib-induced antitumor activity

**Fig. 4:**
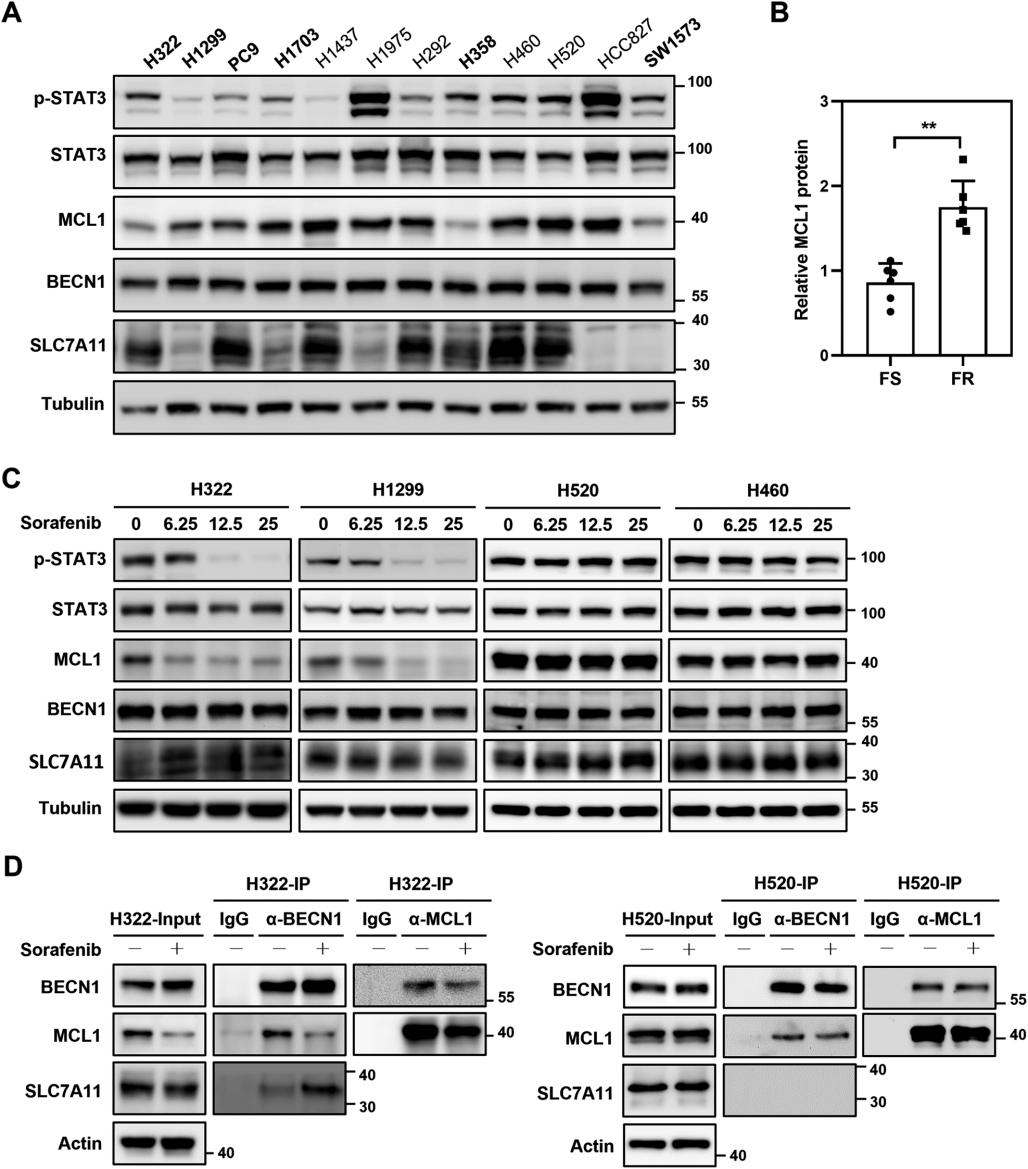
Sorafenib promotes MCL1 downregulation and facilitates the interaction between BECN1 and SLC7A11 in ferroptosis-sensitive NSCLC cells. The endogenous expression levels of STAT3, MCL1, BECN1, and SLC7A11 in a panel of NSCLC cell lines was detected by immunoblotting (**A**). A bar graph illustrates the fold change in MCL1 expression, as determined by immunoblotting in **A**, comparing ferroptosis-sensitive (FS) and -resistant (FR) cell lines (**B**). The expression of STAT3, MCL1, BECN1, and SLC7A11 in response to sorafenib treatment in H322, H1299, H520 and H460 cells was analyzed by immunoblotting (**C**). H322 and H520 cells, with or without sorafenib treatment, were subjected to co-immunoprecipitation and immunoblotting assays using specific antibodies to investigate protein interaction (**D**). **p < 0.01.

The function of Beclin 1 (BECN1) in promoting ferroptosis through its association with and suppression of SLC7A11, the catalytic subunit of the cystine/glutamate antiporter, system Xc^−^, has been uncovered [33]. On the other hand, the interaction between MCL1 and BECN1 sequesters BECN1, thereby protecting cells from the accumulation of reactive oxygen species (ROS) and the unrestrained lipid peroxidation due to system Xc^−^ inhibition [34]. In addition, sorafenib treatment dephosphorylates STAT3 and reduces the expression of MCL1 [35]. We thus hypothesized that MCL1 plays a crucial role in sorafenib-induced ferroptosis in NSCLC, through its expression and interaction with BECN1. We observed a prominent upregulation of MCL1 in ferroptosis-resistant NSCLC cells compared to the ferroptosis-sensitive cells (Figs. 4A and 4B). Interestingly, the expression levels of pSTAT3, BECN1, and SLC7A11 showed no substantial differences (Fig. 4A). Upon sorafenib treatment, there was an effective inhibition of STAT3 activation and a consequent downregulation of MCL1 expression in ferroptosis-sensitive H322 and H1299 cells (Fig. 4C). This treatment, however, had no significant effect on the expression of BECN1 and SLC7A11. Extending these findings to ferroptosis-resistant H520 and H460 cells, we observed that these cells maintained high levels of MCL1 even after sorafenib treatment, which correlated with sustained STAT3 activation (Fig. 4C), contrasting with the sensitive cells. Additionally, results from the co-immunoprecipitation (co-IP) experiments revealed that the decrease in MCL1 levels after sorafenib treatment in sensitive cells led to an increased binding between BECN1 and SLC7A11 (Fig. 4D). Our data suggest that sorafenib treatment downregulates p-STAT3/MCL1, thereby liberating BECN1 from MCL1 sequestration and facilitating the assembly of the BECN1/SLC7A11 complex. The increased binding between BECN1 and SLC7A11contributes to the inhibition of system Xc^−^ and the induction of ferroptosis in NSCLC cells. In contrast, in resistant cells, our co-IP assays showed a consistent interaction between BECN1 and MCL1 and no significant binding between BECN1 and SLC7A11, regardless of sorafenib treatment (Fig. 4D). This lack of interaction may be attributed to the consistently high levels of MCL1 observed in these cells, suggesting a potential mechanism of resistance. These results confirm our hypothesis in the context of both sensitive and resistant cells, highlighting the critical role of MCL1 in modulating key protein interactions in response to sorafenib.

### MCL1 contributes to ferroptosis-resistance in sorafenib-treated NSCLC cells

**Fig. 5:**
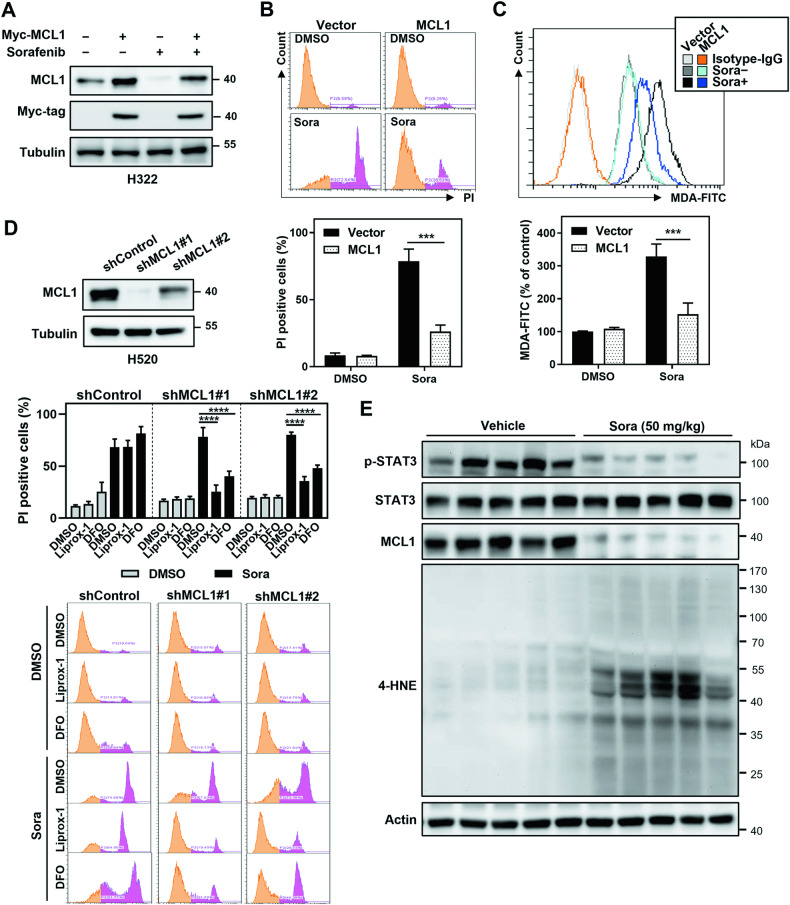
MCL1 confers ferroptosis-resistance to NSCLC cells treated with sorafenib. In ferroptosis-sensitive H322 cells, the efficiency of MCL1 ectopic expression, with or without sorafenib treatment, was assessed by immunoblotting (**A**). The impact of MCL1 ectopic expression on sorafenib-induced ferroptotic cell death was evaluated by flow cytometry after PI-staining (**B**). Additionally, the level of lipid peroxidation, both with and without sorafenib treatment, was determined using the MDA assay (**C**). In ferroptosis-resistant H520 cells, the effect of MCL1 knockdown on sorafenib-induced ferroptotic cell death was evaluated using flow cytometry after PI staining, both in the presence and absence of ferroptosis inhibitors (**D**). The results presented in a bar graph show the mean ± SD from three independent biological replicate experiments (**B**–**D**). The expression levels of STAT3, MCL1, and 4-HNE in H322 xenograft tumor lysates in response to sorafenib were gauged utilizing immunoblotting (**E**). ***p < 0.001; ****p < 0.0001.

To further validate the role of MCL1 in the context of sorafenib-induced ferroptosis in NSCLC cells, we conducted ectopic MCL1 expression experiments in H322 cells (Fig. 5A). Remarkably, we found that elevated MCL1 levels led to a decrease in both sorafenib-associated ferroptotic cell death and the accumulation of malondialdehyde (MDA), a key indicator of lipid peroxidation and ferroptosis (Fig. 5B, C). The findings suggested that MCL1 exerts a protective effect against sorafenib-induced lipid peroxidation and ferroptotic cell death. Conversely, MCL1 knockdown increased the sensitivity of these cells to sorafenib-induced ferroptosis, as evidenced by enhanced cell death which could be rescued by ferroptosis inhibitors (Fig. 5D). This supports the role of MCL1 as a key modulator in ferroptosis resistance. Furthermore, the in vivo experiment using the H322 xenograft model was also conducted. A marked decrease in STAT3 phosphorylation and MCL1 expression, as well as a notable increase in 4-Hydroxynonenal (4-HNE) levels, a final product of lipid peroxidation associated with ferroptosis (Fig. 5E), were observed. These findings strongly suggest that MCL1 contributes to ferroptosis-resistance following sorafenib treatment.

## Discussion

While sorafenib is generally known for its anti-VEGFR and kinases-inhibitory activities, the results presented herein collectively support the notion that ferroptosis is a major driver of sorafenib-induced cell death in NSCLC. Additionally, our findings highlight MCL1’s potential as a ferroptosis-related therapeutic target in NSCLC. The current research also underscores the clinical relevance of triggering ferroptosis pathways to further enhance the efficacy of sorafenib-based therapies.

In addition to its known positive regulators (e.g., TP53, KRAS, VDAC2/3, TFR1, ALOXs) and negative regulators (e.g., SLC7A11, GPX4, NRF2, ATF4, HSPB1) [36], ferroptosis is also regulated by many genes whose roles and associated pathways have not yet been thoroughly elucidated. Activation of STAT3 and upregulation of MCL1 have been previously associated with cancer cell survival and resistance to cell death. However, the role of STAT3 in ferroptosis appears to be somewhat complex and context-dependent. Some studies have suggested that STAT3 can promote ferroptosis by inducing lysosomal membrane permeabilization [37]. Besides, STAT3 has been associated with the regulation of iron metabolism, which is closely linked to ferroptosis susceptibility, during inflammatory conditions [38]. On the contrary, other research has indicated that the interaction between aldo-keto reductase family 1 member B1 (AKR1B1) and STAT3 results in the upregulation of SLC7A11, which promotes ferroptosis resistance in lung cancer [39]. STAT3 activation also can lead to the upregulation of antioxidant defenses and the expression of anti-apoptotic genes [40]. Our study described herein establishes a pivotal role of the STAT3/MCL1 axis in sorafenib-induced ferroptosis in NSCLC. We observed a significant decrease in STAT3 activation and MCL1 expression upon in vivo sorafenib treatment, accompanied by an increase in ferroptosis-associated 4-HNE levels (Fig. 5D). Inhibiting STAT3 activation by sorafenib may downregulate MCL1, but high expression of MCL1 does not necessarily correlate positively with STAT3 activation (Fig. 4A, B). In addition to the STAT3 pathway, the expression of MCL1 is also influenced by various other cell signaling pathways, such as PI3K/AKT, MAPK, and others [41]. Furthermore, the complexity of cell signaling pathways and the diversity of intracellular and extracellular environments may lead to a lack of strict positive correlation between the high expression of MCL1 and the activation of STAT3. Nevertheless, our results suggest that upregulation of MCL1 competes with SLC7A11 for BECN1 binding and plays a key role in ferroptosis-resistance. Therefore, targeting MCL1 could represent a promising avenue to sensitize NSCLC cells to ferroptosis and overcome therapeutic resistance caused by ferroptosis inhibition.

In cancer therapy, resistance to chemotherapy, radiation therapy, targeted therapies, and immunotherapies remains a major challenge that often leads to treatment failure, disease recurrence, and poor clinical outcomes. In recent years, MCL1 upregulation has been implicated in therapeutic resistance in various types of cancer and other diseases [42]. As an anti-apoptotic protein, MCL1 acts as a sentinel of survival, promoting cell survival and inhibiting apoptosis in the presence of cytotoxic stresses induced by therapeutic agents [43]. Through its interactions with other members of the Bcl-2 family, MCL1 stabilizes the mitochondrial outer membrane, blocking the release of cytochrome c and inhibiting the onset of the apoptotic cascade [44]. Consequently, cancer cells with elevated MCL1 expression are endowed with a survival advantage, counteracting the cytotoxic effects of therapies and leading to therapeutic resistance. MCL1 is also intricately involved in regulating autophagy and ferroptosis [34, 45], two additional cell death pathways with emerging attention in cancer biology. In certain cellular contexts, MCL1 can either inhibit or be targeted by autophagy, depending on the cellular stimuli, leading to diverse outcomes in cell fate determination [44]. Similarly, MCL1 upregulation has been observed to protect cancer cells from undergoing ferroptosis, characterized by lipid peroxidation. In our study, ectopic MCL1 expression in H322 cells alleviated sorafenib-induced ferroptotic cell death and MDA accumulation, suggesting its role as a ferroptosis suppressor. Notably, the intricate crosstalk between STAT3, MCL1, and the autophagy regulator BECN1 indicates a complex regulatory network influencing ferroptosis sensitivity. Our results indicate that MCL1 upregulation, at least in part through STAT3 activation, plays a critical role in mediating the ferroptosis resistance to sorafenib. The crosstalk among multiple cell death pathways centered around MCL1 underscores its capacity to impact therapeutic responses through diverse mechanisms. As sorafenib has already received clinical approval and its safety profile is well-established, our findings provide a robust foundation for future endeavors aimed at harnessing its ferroptotic-inducing activity for NSCLC treatment.

In summary, our study reveals that, following sorafenib treatment, ferroptosis is the predominant form of cell death in a significant subset of NSCLC cell types. The suppression of MCL1 due to sorafenib treatment unleashes BECN1, facilitating its interaction with and inhibition of SLC7A11, leading to ferroptosis in NSCLC cells. Our data not only suggests MCL1’s role in conferring resistance to ferroptosis but also positions MCL1 as a target to initiate ferroptosis, enhancing the therapeutic efficacy against NSCLC. By inhibiting the STAT3/MCL1 signaling axis and predisposing NSCLC cells to ferroptosis, our study provides significant insights for the development of innovative combinations to treat advanced NSCLC patients.

## Materials and methods

### Reagents, chemicals, and antibodies

All reagents, chemicals, and antibodies used in this study can be found in Table 2.

**Table 2:** Reagents, chemicals, assay kits, and antibodies used in this study.

Item	Supplier	Catalog number
DMEM	Thermo Fisher Scientific	12100-046
Opti-MEM	Thermo Fisher Scientific	31985-070
10× Trpsin-EDTA	Thermo Fisher Scientific	15400-054
10× phosphate-buffered saline	Thermo Fisher Scientific	21600-069
Hanks’ Balanced Salt Solution	Thermo Fisher Scientific	14025-076
100× PSA	Biological Industries	BII03-033-1B
Fetal bovine serum	Biological Industries	04-001-1A
EZPCR Mycoplasma Test Kit	Biological Industries	20-700-20
Human pCMV6-MCL1-Myc-DDK plasmid	OriGene	RC200521
Lipofectamine 3000 reagent	Thermo Fisher Scientific	L3000-015
Sorafenib	MedChemExpress	HY-10201A
Liproxstatin-1 (Liprox-1)	TargetMol	T2376
Deferoxamine mesylae (DFO)	TargetMol	T1637
β-Mercaptoethanol (2-ME)	Sigma-Aldrich	M3148
L-glutathione reduced (GSH)	Sigma-Aldrich	G4251
C11-BODIPY probe	Thermo Fisher Scientific	D3861
MDA assay kit	Abcam	ab118970
GSH assay kit	Abcam	ab239709
Glutamate-Glo™ Assay kit	Promega	J7021
p-STAT3 (Y705) rabbit monoclonal Ab	Cell Signaling Technology	#9145
STAT3 mouse monoclonal IgG	Cell Signaling Technology	#9139
MCL1 rabbit polyclonal IgG	Cell Signaling Technology	#4572
MCL1 rabbit monoclonal IgG for IP	Cell Signaling Technology	#94296
SLC7A11 rabbit monoclonal IgG	Cell Signaling Technology	#12691
BECN1 mouse monoclonal IgG	Cell Signaling Technology	#4122
BECN1 rabbit monoclonal IgG for IP	Cell Signaling Technology	#3495
4-Hydroxynonenal rabbit polyclonal IgG	Abcam	ab46545
Myc-tag mouse monoclonal IgG	Cell Signaling Technology	#2276
β-tubulin rabbit monoclonal IgG	Cell Signaling Technology	#2128
β-actin mouse monoclonal IgG	Proteintech	66009-1-lg
Goat anti-rabbit IgG-HRP	Santa Cruz Biotechnology	sc-2004
Goat anti-mouse IgG-HRP	Santa Cruz Biotechnology	sc-2005
Normal mouse IgG1	Santa Cruz Biotechnology	sc-3877
Normal rabbit IgG	Santa Cruz Biotechnology	sc-2027

### Cell culture and treatments

The H322, H1299, PC9, H1703, H1437, H1975, H292, H358, H460, H520, HCC827, and SW1573 cell lines were procured from the American Type Culture Collection (ATCC). All cell lines were cultured in RPMI-1640 medium supplemented with 10% fetal bovine serum, 2 mM L-glutamine, 10 mM HEPES, 4 500 mg/L glucose, 1 mM sodium pyruvate, and 1 500 mg/L sodium bicarbonate. The cells were maintained in a 37 °C humidified incubator with 5% CO2 in the air. Regular screening for mycoplasma contamination was performed using the EZPCR Mycoplasma Test Kit (Biological Industries, Beit-Haemek, Israel). For transient transfection, Lipofectamine 3000 reagent (Invitrogen, CA, USA) was used following the manufacturer’s instructions. After 48 h of transfection, the cells were treated with specific concentrations and durations of drug as indicated in the study.

### Lipid ROS assay

The C11-BODIPY probe was utilized to assess the production of lipid ROS. Briefly, cells were seeded in 6-well plates at a density of 3 × 10^5^ cells per well and then treated with the specified drug concentrations. After treatment, the cells were harvested through trypsinization and stained with C11-BODIPY (2.5 µM) at 37 °C for 20 min. The stained cells were subsequently resuspended in 500 µL of Hank’s Balanced Salt Solution and subjected to flow cytometry analyses using a CytoFLEX flow cytometer (Beckman Coulter, Brea, CA, USA). The mean fluorescence values from a minimum of 10,000 cells were calculated using CytExpert 2.4 software (Beckman Coulter, CA, USA).

### Malondialdehyde, glutathione, and glutamate release assays

The Malondialdehyde (MDA) Assay kit and Glutathione Assay kit were employed to measure lipid peroxidation and the concentration of total glutathione (GSH) in cell culture and tissue extracts, respectively. For assessing glutamate released from cells into the extracellular medium, the Glutamate-Glo™ Assay kit was utilized according to the manufacturer’s instructions. To account for potential variations in cell numbers, each assay was normalized to total protein concentration, which was determined using the bicinchoninic acid (BCA) assay at the experiment’s endpoint. The resulting values were then expressed as a percentage relative to the control group.

### Propidium iodide (PI)-staining

The cells were plated in a 6-well plate at a density of 1.5–2 × 10^5^ cells per well. After 48 h of treatment, the cells were collected, rinsed with ice-cold PBS, and stained with propidium iodide (PI) solution (2 μg/ml in PBS) for 15 min at 37 °C. Subsequently, the cells were washed with 0.2% PBST and resuspended in 500 μl of PBS, and flow cytometry analysis was performed within 1 h.

### Xenograft model

Male BALB/c nude mice (4 weeks old) were obtained from the National Laboratory Animal Center (Taipei, Taiwan). Every experiment involving these mice were carried out following the protocol (no. IACUC-110C-003) sanctioned by the Institutional Laboratory Animal Care and Use Committee of Cardinal Tien Hospital on 03/Feb/2021. For our tumor xenograft studies, each mouse was injected subcutaneously on their dorsal flank with 5 × 10^6^ H322 cells in a 0.1 mL solution of PBS mixed with 50% Matrigel (BD Biosciences, Bedford, MA, USA). When tumor sizes reached approximately 400 mm^3^, the mice were randomized into two groups without blinding to administer sorafenib (50 mg/kg) or vehicle (ethanol/Cremophor EL/water = 1:1:6) orally once daily for two weeks (*n* = 8 for each group). Throughout the study, the body weights and tumor sizes of the mice were measured and recorded twice weekly.

### Immunoblotting

Cell lysates were prepared using a RIPA buffer consisting of 150 mM NaCl, 50 mM Tris-HCl, 0.1% SDS, 1% Triton X-100, 0.5% sodium deoxycholate, 2 mM EDTA, 50 mM sodium fluoride, 0.5 mM dithiothreitol, and 1× protease/phosphatase inhibitor cocktail (Roche Diagnostic). After centrifugation at 13,000 rpm for 10 min, the protein lysates were collected, and their concentrations were determined using the BCA assay. Equal amounts of protein were denatured at 95 °C for 5 min and loaded onto 4% stacking gels, followed by separation through SDS electrophoresis on 8% or 10% gels. The separated proteins were then transferred to nitrocellulose membranes using semi-dry electroblotting. Subsequently, the membranes were blocked in Tris-buffered saline containing 0.1% Tween 20 and 5% non-fat milk and incubated overnight at 4 °C with the appropriate primary antibodies. After washing the membranes, they were incubated with horseradish peroxidase-conjugated secondary antibodies, washed again, and developed using enhanced chemiluminescence. The visualized bands were captured by an Imaging System, and densitometry analysis was performed to quantify the band intensities.

### Co-immunoprecipitation

Cells were washed and lysed in NP40-buffer, which consisted of 50 mM Tris-HCl (pH 7.4), 150 mM NaCl, 2 mM EDTA, and 0.1% NP40, supplemented with 1× protease/phosphatase inhibitor cocktail. The protein lysates were collected by centrifugation at 13,000 rpm for 15 min at 4 °C after 20 min of incubation on ice. To immunoprecipitate BECN1 or MCL1, 1 μg of corresponding antibodies was added to each lysate, followed by an incubation period of 60 min at 4 °C on a rotating wheel. Subsequently, 20 μl of Protein A agarose was added, and the reaction was further incubated for 60 min. The agarose beads were collected by centrifugation, subjected to four washes with 1 ml of NP40-buffer each time, and then boiled. The proteins were finally analyzed by immunoblotting.

### Statistical analysis

The results were expressed as mean ± standard deviation (SD) from the indicated number of independent experiments. For experiments involving two groups, a Student’s *t* test was employed to assess significant differences. In cases where more than two groups were studied, an ANOVA was initially used, followed by the multi-range Dunett’s *t*-test. For experiments with effective matching, a paired Student’s *t* test or repeated ANOVA was utilized. Statistical significance was determined at *p* < 0.05 level for all mean differences.

### Supplementary information

Original Data

## Data Availability

All the data substantiating the results of this study are accessible within the article or can be obtained from the corresponding author upon reasonable inquiry.
