# Supplementary figures and images for: MCL1 inhibition: a promising approach to augment the efficacy of sorafenib in NSCLC through ferroptosis induction
Source: Cell Death Discov. 2024 Mar 14;10:137. doi: 10.1038/s41420-024-01908-5 (PMC10940654; doi:10.1038/s41420-024-01908-5)

Fig. 4A

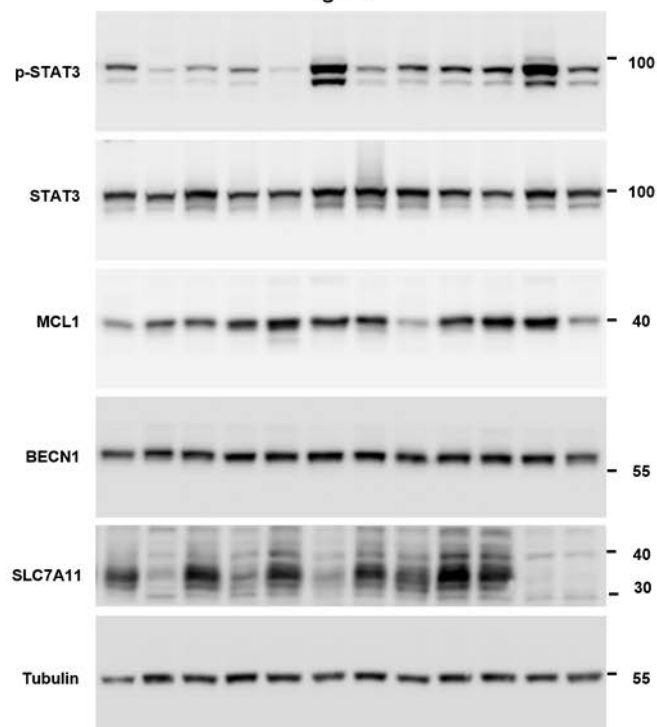

Fig. 4C

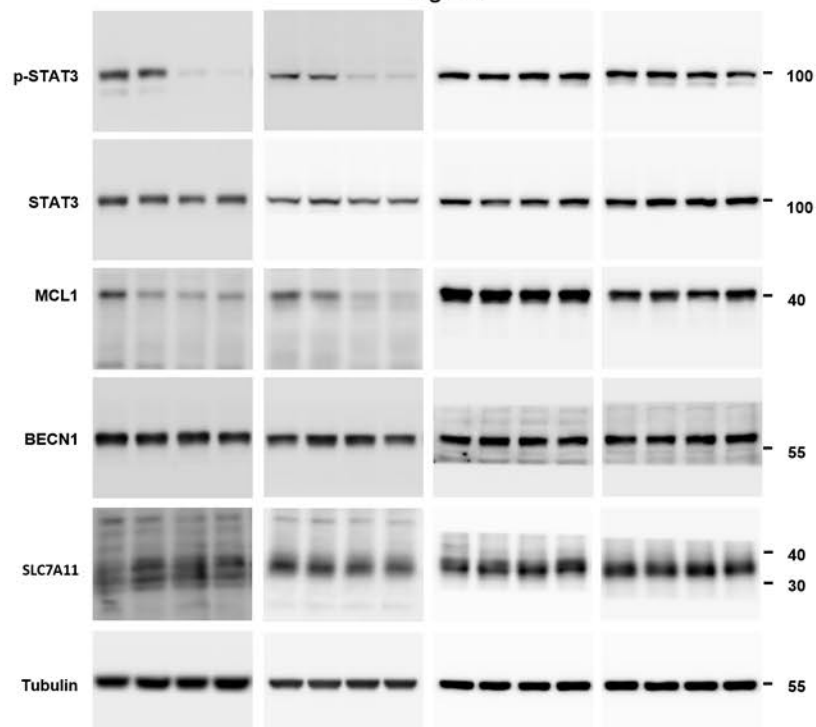

Fig. 4D

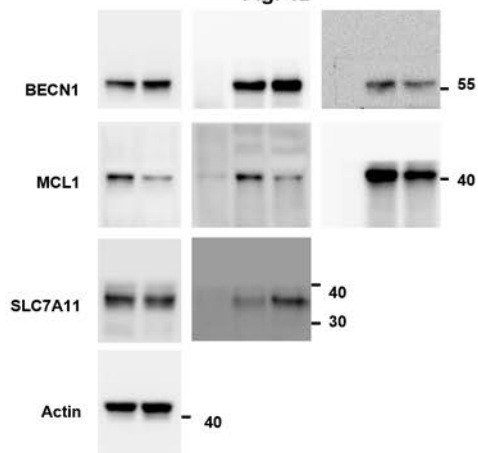

Fig. 5A

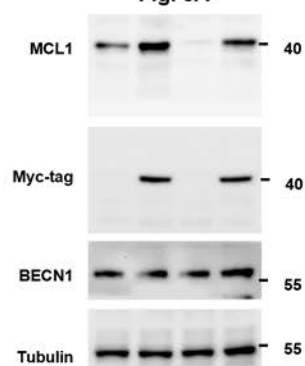

Fig. 5D

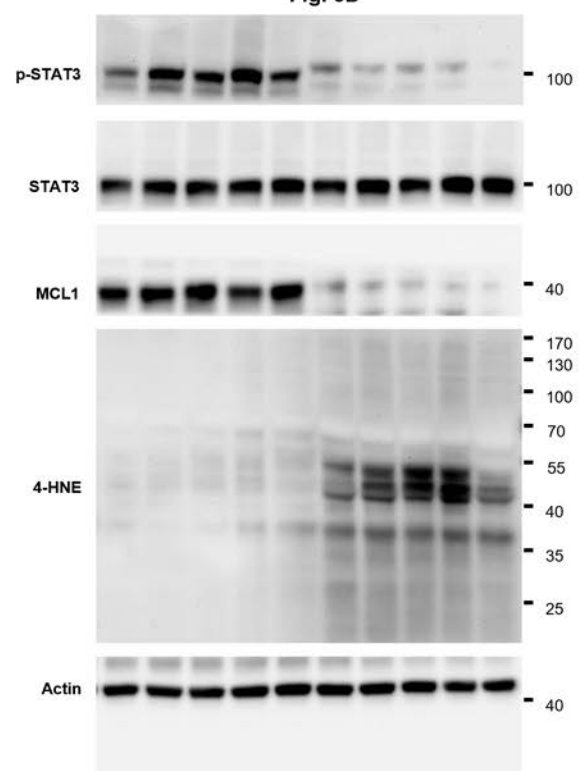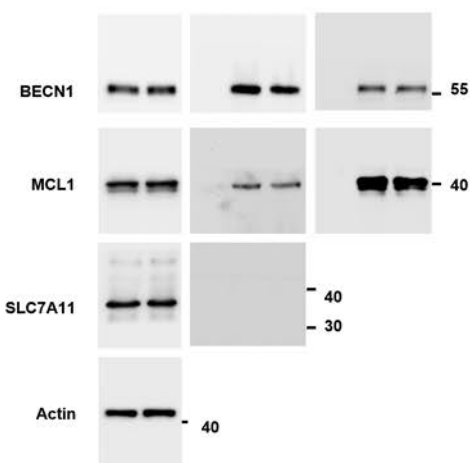

Supplement: Supplementary file 1 — Original Data [file 41420_2024_1908_MOESM1_ESM.pdf]
